# Supplementary material for: Non-linear and Interaction Analyses of Biomarkers for Organ Dysfunctions as Predictive Markers for Sepsis: A Nationwide Retrospective Study
Source: J Pers Med. 2022 Jan 4;12(1):44. doi: 10.3390/jpm12010044 (PMC8778987; doi:10.3390/jpm12010044)
Supplement: Supplementary file 1 [file jpm-12-00044-s001.zip › jpm-1470177-supplementary.pdf]

Journal of Personalized Medicine

MDPI

Supplementary Materials

Table S1. Definitions of the presence of infection, organ dysfunction, and comorbidities.

Group	Code Type	Code
Infection	ICD 10 codes in the primary or trigger-for-hospitalization diagnosis	A01–A02.0, A03–A09.9, A19–A20.3, A21–A21.3, A22–A22.2, A23–A24.0, A25–A26.0, A27–A28.1, A31–A32.12, A36–A39, A39.1–A39.3, A42–A42.2, A43–A46.0, A48–A49.9, A59–A59.9, A65–A65.0, A69–A69.1, A74, A74.8–A75.9, A77–A81.9, A83–A96.9, A98–B00.59, B01–B10.89, B25–B27.99, B29.4, B33–B34.9, B37–B37.6, B38–B50.9, B54–B55, B55.1–B55.9, B58–B60.8, B64, B67–B67.99, B91, B95–B99.9, G00–G08.0, G14–G14.6, H05.01–H05.039, H60.2–H60.23, H70.0–H70.009, I00, I02, I02.9, I26.01–I26.09, I26.90–I26.99, I33–I33.9, I38–I39.9, I40.0–I40.9, I76, I96–I96.9, I98.1, J01–J06.9, J09–J22.9, J36–J36.0, J39.0–J39.1, J85–J86.9, K35–K37.9, K57–K57.93, K61–K61.4, K63.0–K63.1, K65–K65.9, K67.8, K75.0–K75.1, K75.3, K76.3, K77.0, K81.0, K81.2, K83.0, K95.01, K95.81, L02–L08.9, M00–M02.9, M86–M86.9, M89.6–M89.69, N10–N10.9, N15.1–N15.9, N30–N30.91, N39.0, N41.0, N41.2–N41.3, N45–N45.9, N70–N77.8, R78.81, T80.2–T80.29, T81.4, T82.6–T82.7, T83.5, T83.6, T84.5–T84.7, T85.7, T88.0, U04
Organ dysfunction, IHME [11]	ICD 10 codes in the primary, concomitant or complication diagnoses	D65–D65.9, D69.5–D69.59, E87.2–E87.99, G93.4–G93.49, I46–I46.9, I95.1–I95.9, J80–J80.9, J95.2–J95.3, J96–J96.92, K72–K72.91, N00–N01.9, N17–N17.9, R09.02, R09.2, R40.0–R40.4, R41.82, R55–R55.0, R57–R57.9
Organ dysfunction, Angus [14]	ICD 10 codes in the primary, concomitant or complication diagnoses	I95, R57, Z99.1, D65, D68.9, D69.5, D69.6, F05.0, F05.8, F05.9, G63.1, G93.4, R41, K72.0, K72.9, K76.2, K76.3, N17, N99.0
Organ dysfunction, Martin [15]	ICD 10 codes in the primary, concomitant or complication diagnoses	I95.1, I95.9, R03.1, R57, J95.1, J95.2, J80, R06.0, R06.3, R06.4, R06.8, R09.2, J96.0, J96.9, D65, D68.9, D69.3, D69.4, D69.5, D69.6, F05.0, F05.8, F05.9, G93.1, G93.4, R40, R41.8, R41.0, N00, N01, N17, N19, Z49.1, K72.0, K72.9, K76.2, E87.2
Charlson Comorbidity Index		
Myocardial infarction	ICD 10 codes in the concomitant or complication diagnoses	I21.x, I22.x, I25.2
Congestive heart failure	ICD 10 codes in the concomitant or complication diagnoses	I09.9, I11.0, I13.0, I13.2, I25.5, I42.0, I42.5–I42.9, I43.x, I50.x, P29.0
Peripheral vascular disease	ICD 10 codes in the concomitant or complication diagnoses	I70.x, I71.x, I73.1, I73.8, I73.9, I77.1, I79.0, I79.2, K55.1, K55.8, K55.9, Z95.8, Z95.9
Cerebrovascular disease	ICD 10 codes in the concomitant or complication diagnoses	G45.x, G46.x, H34.0, I60.x–I69.x
Dementia	ICD 10 codes in the concomitant or complication diagnoses	F00.x–F03.x, F05.1, G30.x, G31.1
Chronic pulmonary disease	ICD 10 codes in the concomitant or complication diagnoses	I27.8, I27.9, J40.x–J47.x, J60.x–J67.x, J68.4, J70.1, J70.3
Rheumatic disease	ICD 10 codes in the concomitant or complication diagnoses	M05.x, M06.x, M31.5, M32.x–M34.x, M35.1, M35.3, M36.0
Peptic ulcer disease	ICD 10 codes in the concomitant or complication diagnoses	K25.x–K28.x
Mild liver disease	ICD 10 codes in the concomitant or complication diagnoses	B18.x, K70.0–K70.3, K70.9, K71.3–K71.5, K71.7, K73.x, K74.x, K76.0, K76.2–K76.4, K76.8, K76.9, Z94.4
Diabetes without chronic complication	ICD 10 codes in the concomitant or complication diagnoses	E10.0, E10.1, E10.6, E10.8, E10.9, E11.0, E11.1, E11.6, E11.8, E11.9, E12.0, E12.1, E12.6, E12.8, E12.9, E13.0, E13.1, E13.6, E13.8, E13.9, E14.0, E14.1, E14.6, E14.8, E14.9
Diabetes with chronic complication	ICD 10 codes in the concomitant or complication diagnoses	E10.2–E10.5, E10.7, E11.2–E11.5, E11.7, E12.2–E12.5, E12.7, E13.2– E13.5, E13.7, E14.2–E14.5, E14.7
Hemiplegia or paraplegia	ICD 10 codes in the concomitant or complication diagnoses	G04.1, G11.4, G80.1, G80.2, G81.x, G82.x, G83.0–G83.4, G83.9
Renal disease	ICD 10 codes in the concomitant or complication diagnoses	I12.0, I13.1, N03.2–N03.7, N05.2–N05.7, N18.x, N19.x, N25.0, Z49.0–Z49.2, Z94.0, Z99.2
Any malignancy, including lymphoma and leukemia, except malignant neoplasm of skin	ICD 10 codes in the concomitant or complication diagnoses	C00.x–C26.x, C30.x–C34.x, C37.x–C41.x, C43.x, C45.x–C58.x, C60.x–C76.x, C81.x–C85.x, C88.x, C90.x–C97.x
Moderate or severe liver disease	ICD 10 codes in the concomitant or complication diagnoses	I85.0, I85.9, I86.4, I98.2, K70.4, K71.1, K72.1, K72.9, K76.5, K76.6, K76.7

Metastatic solid tumor	ICD 10 codes in the concomitant or complication diagnoses	C77.x–C80.x
AIDS/HIV	ICD 10 codes in the concomitant or complication diagnoses	B20.x–B22.x, B24.x

ICD-10: International Classification of Diseases Tenth Revision; IHME: Institute for Health Metrics and Evaluation; AIDS/HIV: Acquired Immune Deficiency Syndrome/Human Immunodeficiency Virus.

Table S2. Modified definitions for calculation of SOFA subscores.

SOFA Subscore	1	2	3	4
Respiratory	Oxygenation	High-flow nasal cannula oxygen or non-invasive positive pressure ventilation	Mechanical ventilation	N.A.
Coagulation	Platelets <150 × 10^3/μL	Platelets <100 × 10^3/μL	Platelets <50 × 10^3/μL	Platelets <20 × 10^3/μL
Liver	Bilirubin >1.2 mg/dL	Bilirubin >2.0 mg/dL	Bilirubin >6.0 mg/dL	Bilirubin >12.0 mg/dL
Cardiovascular	N.A.	DOA <5 or any DOB	DOA 5.1 to 15, AD ≤0.1, or NAD ≤0.1	DOA >15, AD >0.1, or NAD >0.1
Neurological	Japan Coma Scale = 1, 2, or 3	Japan Coma Scale = 10 or 20	Japan Coma Scale = 30 or 100	Japan Coma Scale = 200 or 300
Renal	Creatinine >1.2 mg/dL	Creatinine >2.0 mg/dL	Creatinine >3.5 mg/dL	Creatinine >5.0 mg/dL

SOFA: Sequential Organ Failure Assessment; AD: Adrenaline, DOA: Dopamine, DOB: Dobutamine, NAD: Noradrenaline, N.A.: Not Applicable.

Table S3. Percentiles of knots and thresholds of biomarkers in the restricted cubic spline analyses.

Biomarker	Number of Knots	Threshold Percentiles			
Platelet count (10^3/μL)	4	6.1	13.4	19.2	34.1
		5	35	65	95
Bilirubin (mg/dL)	4	0.3	0.6	1.7	4.6
		5	35	65	95
Creatinine (mg/dL)	3	0.57	1.06	3.21	
		10	50	90	
SOFA total	3	3	4	7	
		10	50	90	

SOFA: Sequential Organ Failure Assessment.

Table S4. Univariate and multivariate logistic regression analyses.

	Univariate Analyses			Multivariate Analyses		
**SOFA Subscores**	**Odds Ratio**	**95% CI**	***p* Value**	**Odds Ratio**	**95% CI**	***p* Value**
Neurological score	1.71	1.66–1.76	<0.001	1.62	1.57–1.67	<0.001
Cardiovascular score	1.58	1.52–1.64	<0.001	1.3	1.24–1.37	<0.001
Coagulation score	1.16	1.13–1.2	<0.001	1.36	1.31–1.41	<0.001
Hepatic score	0.76	0.73–0.79	<0.001	1.04	0.99–1.08	0.123
Renal score	1.11	1.09–1.14	<0.001	1.24	1.2–1.27	<0.001

SOFA: Sequential Organ Failure Assessment, CI: Confidence Interval.

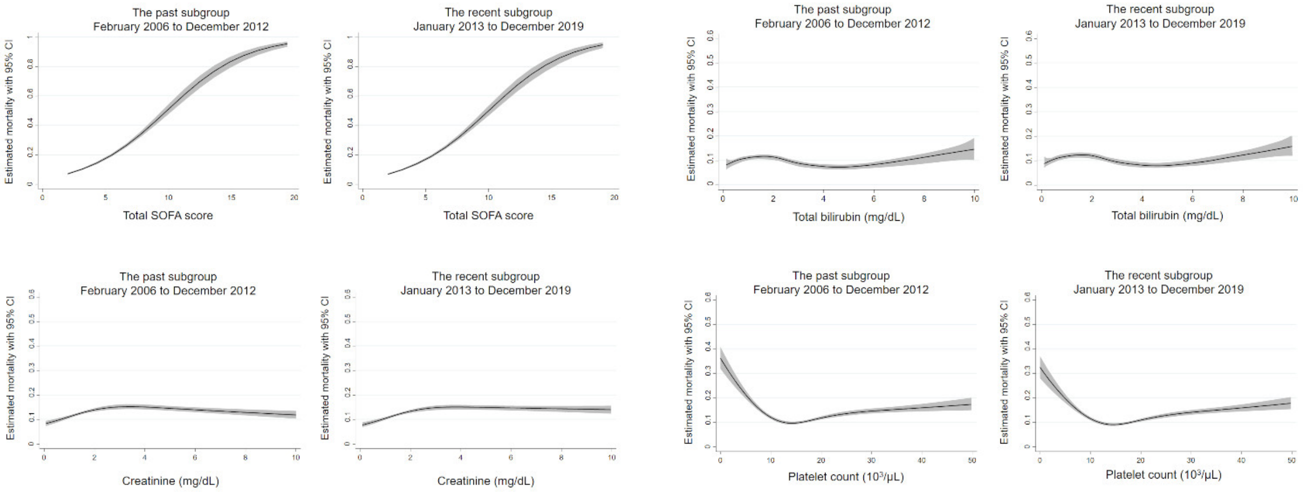

Figure S1. Restricted cubic spline analyses in the two subgroups based on the time of hospital admission (the earlier group: 1 February 2006 to 31 December 2012 and the recent group: 1 January 2013 to 31 December 2019). The black line represents the fitted line of the association between biomarkers and the estimated mortality risk, and the shaded region represents the 95% confidence interval. Total SOFA score was the sum of the five SOFA subscores except for the respiratory subscore. SOFA: Sequential Organ Failure Assessment, CI: Confidence Interval.

Table S5. STROBE Statement—Checklist of items that should be included in reports of cohort studies.

	Item No.	Recommendation	Page No.
Title and abstract	1	Indicate the study's design with a commonly used term in the title or the abstract	1
		Provide in the abstract an informative and balanced summary of what was done and what was found	1
		Introduction	
Background/rationale	2	Explain the scientific background and rationale for the investigation being reported	1–2
Objectives	3	State specific objectives, including any prespecified hypotheses	2
		Methods	
Study design	4	Present key elements of study design early in the paper	2
Setting	5	Describe the setting, locations, and relevant dates, including periods of recruitment, exposure, follow-up, and data collection	2
Participants	6	Give the eligibility criteria, and the sources and methods of selection of participants. Describe methods of follow-up	2
		For matched studies, give matching criteria and number of exposed and unexposed	N.A.
Variables	7	Clearly define all outcomes, exposures, predictors, potential confounders, and effect modifiers. Give diagnostic criteria, if applicable	3
Data sources/ measurement	8*	For each variable of interest, give sources of data and details of methods of assessment (measurement). Describe comparability of assessment methods if there is more than one group	3
Bias	9	Describe any efforts to address potential sources of bias	3–4
Study size	10	Explain how the study size was arrived at	N.A.
Quantitative variables	11	Explain how quantitative variables were handled in the analyses. If applicable, describe which groupings were chosen and why	3
Statistical methods	12	Describe all statistical methods, including those used to control for confounding	3–4
		Describe any methods used to examine subgroups and interactions	3–4
		Explain how missing data were addressed	4
		If applicable, explain how loss to follow-up was addressed	N.A.
		Describe any sensitivity analyses	4
		Results	
Participants	13*	Report numbers of individuals at each stage of study—e.g., numbers potentially eligible, examined for eligibility, confirmed eligible, included in the study, completing follow-up, and analyzed	4
		Give reasons for non-participation at each stage	4
		Consider use of a flow diagram	Figure 1
Descriptive data	14*	Give characteristics of study participants (e.g., demographic, clinical, social) and information on exposures and potential confounders	5
		Indicate number of participants with missing data for each variable of interest	Figure 3
		Summarise follow-up time (e.g., average and total amount)	N.A.
Outcome data	15*	Report numbers of outcome events or summary measures over time	4
Main results	16	Give unadjusted estimates and, if applicable, confounder-adjusted estimates and their precision (e.g., 95% confidence interval). Make clear which confounders were adjusted for and why they were included	6
		Report category boundaries when continuous variables were categorized	3, 7
		If relevant, consider translating estimates of relative risk into absolute risk for a meaningful time period	N.A.
Other analyses	17	Report other analyses done—e.g., analyses of subgroups and interactions, and sensitivity analyses	Figure 4
		Discussion	
Key results	18	Summarise key results with reference to study objectives	8
Limitations	19	Discuss limitations of the study, taking into account sources of potential bias or imprecision. Discuss both direction and magnitude of any potential bias	10
Interpretation	20	Give a cautious overall interpretation of results considering objectives, limitations, multiplicity of analyses, results from similar studies, and other relevant evidence	9
Generalizability	21	Discuss the generalisability (external validity) of the study results	9
		Other information	
Funding	22	Give the source of funding and the role of the funders for the present study and, if applicable, for the original study on which the present article is based	10

*Give information separately for exposed and unexposed groups. Note: An Explanation and Elaboration article discusses each checklist item and gives methodological background and published examples of transparent reporting. The STROBE checklist is best used in conjunction with this article (freely available on the Web sites of PLoS Medicine at

http://www.plosmedicine.org/, Annals of Internal Medicine at http://www.annals.org/, and Epidemiology at http://www.epidem.com/). Information on the STROBE Initiative is available at http://www.strobe-statement.org. STROBE: Strengthening the Reporting of Observational Studies in Epidemiology, N.A.: Not Applicable.
